# Familial, constitutional, and combined idiopathic short stature: longitudinal growth patterns and pubertal effects

**DOI:** 10.3389/fped.2026.1783065

**Published:** 2026-03-12

**Authors:** Erkut Gürlek, Sirmen Kızılcan Çetin, Elif Özsu, Zehra Aycan, Merih Berberoğlu, Zeynep Şıklar

**Affiliations:** 1 Department of Pediatrics, Ankara University School of Medicine, Ankara, Türkiye; 2 Department of Pediatric Endocrinology, Ankara University School of Medicine, Ankara, Türkiye

**Keywords:** bone age, constitutional delay, familial short stature, growth velocity, idiopathic short stature, puberty

## Abstract

**Background:**

Pathological causes account for approximately 15%–20% of short-stature cases, whereas about 80% of short-statured children have no identifiable underlying etiology and are classified as idiopathic short stature (ISS). ISS represents a highly heterogeneous group, and ongoing debates persist due to the limited availability of observational data and advances in genetic research. Despite its high prevalence, long-term auxological data comparing familial, constitutional, and combined variants across pubertal stages remain limited.

**Objectives:**

Our study aimed to characterize the clinical and laboratory features at presentation and to evaluate the longitudinal growth patterns of children initially diagnosed with ISS.

**Methods:**

A retrospective cohort of 171 children with ISS (46.2% female) was analyzed. Participants were classified as prepubertal (Group 1; *n* = 121) and pubertal (Group 2; *n* = 50), each further subdivided into familial (a), constitutional (b), and combined (c) subgroups. Anthropometric, familial, and biochemical parameters were assessed at presentation and final follow-up. Standard deviation scores (SDS) were calculated based on national growth references. Intergroup comparisons were performed using ANOVA or the Kruskal–Wallis test with *post hoc* corrections. Statistical significance was accepted at *p* < 0.05.

**Results:**

Mean age at first evaluation was 7.94 ± 4.46 years; mean height SDS was −2.45 ± 0.34 with proportionate body proportions and normal birth parameters. Bone age averaged 6.57 ± 4.28 years (≈1.4-year delay). The prepubertal/pubertal distribution was 121 (70.8%) vs. 50 (29.2%); combined phenotypes comprised 53.5% of the cohort. Over 1.85 ± 1.40 years of follow-up, mean ΔHeight SDS was +0.35 ± 0.56; pubertal subgroups, particularly 2b and 2c, showed the most significant gains (ΔHeight SDS +0.58 and +0.53; both *p* < 0.001 vs. prepubertal). ΔHeight SDS correlated positively with baseline bone-age delay (*p* < 0.001) and inversely with age (*p* = 0.002). Growth velocity was normal in all. BMI SDS rose modestly overall (−0.63 ± 0.99 to −0.49 ± 0.92; *p* = 0.04) and remained below +2 SDS in all cases.

**Conclusions:**

ISS subtypes display distinct auxological courses. Bone-age delay is a key predictor of subsequent catch-up growth, most evident in pubertal CDGP and combined phenotypes. Given the high rate of spontaneous improvement, especially after pubertal onset, careful longitudinal monitoring should precede pharmacologic therapy.

## What is known

Idiopathic short stature (ISS) is a heterogeneous diagnosis including familial, constitutional, and combined forms.
Bone-age delay is a key determinant of growth potential, especially in constitutional delay of growth and puberty (CDGP).
Most children with ISS maintain normal growth velocity.

## What is New

Combined ISS phenotypes show taller parental heights and higher target-height SDS than classical familial/constitutional groups.
Pubertal constitutional and combined subgroups demonstrate the greatest spontaneous catch-up (ΔHeight SDS >0.5).
Baseline bone-age delay strongly predicts longitudinal height gain, supporting conservative follow-up before rhGH use.

## Introduction

Short stature is defined as a height below the 3rd percentile or more than 2 standard deviations (SD) below the mean for age and sex on growth charts. Severe short stature refers to height below −3 SD, while growth retardation indicates subnormal growth velocity before short stature develops. Idiopathic short stature (ISS) is a diagnosis of exclusion applied to children whose height falls below −2 SD for age and sex, without systemic disease, endocrine deficiency, or chromosomal abnormality (1). It encompasses a clinically heterogeneous population, including familial short stature (FSS), constitutional delay of growth and puberty (CDGP), and combined forms (1).

The differentiation between FSS, CDGP, and combined ISS is crucial for predicting growth outcomes and determining the need for therapeutic intervention. FSS is primarily influenced by genetic potential, reflected by short parental heights and normal growth velocity. In contrast, CDGP involves transient deceleration of growth and delayed bone maturation, with eventual achievement of normal adult height. The combined form represents an overlapping phenotype with both genetic predisposition and delayed maturation.

While several studies have described these subtypes individually, few have provided a comprehensive comparative analysis integrating prepubertal and pubertal stages within the same cohort. Moreover, most published data lack longitudinal follow-up beyond the initial evaluation, limiting insights into dynamic changes in growth velocity and bone age progression (1, 2). Understanding these scenarios is vital for both clinical counseling and therapeutic planning. For instance, identifying those with delayed but self-limited growth can prevent unnecessary treatment with recombinant growth hormone (rhGH), whereas recognizing patients with persistent short stature despite adequate bone maturation may guide earlier intervention (1, 2).

This study aims to provide a detailed auxological profile of children with ISS across both prepubertal and pubertal stages, evaluating familial, constitutional, and combined variants. We sought to analyze growth parameters, bone age, parental contribution, and follow-up outcomes, highlighting developmental transitions and catch-up growth patterns within a large cohort.

## Methods

### Study design and participants

This retrospective, cross-sectional, and longitudinal study included all patients diagnosed with ISS who were followed in our Pediatric Endocrinology Department (January 1, 2010–December 31, 2022).

Height below −2.0 SDS according to Turkish national standards (4);
Normal thyroid function, growth hormone axis, and karyotype (in girls);
Absence of chronic systemic disease, skeletal dysplasia, or syndromic features.

Only patients with a minimum follow-up duration of at least 6 months were included in the analysis to ensure reliable assessment of longitudinal changes in height SDS. Exclusion criteria included growth hormone deficiency, chronic malnutrition, systemic illness, mental disease and use of medications affecting growth.

A normal GH axis was defined based on: normal growth velocity, absence of clinical features of GH deficiency. IGF-1 and IGFBP-3 values interpreted in clinical context. Low IGF-1 alone was not considered diagnostic of GH deficiency.

### Group classification

Pubertal status was defined by Tanner staging (5): testicular volume ≥4 mL in boys and breast development ≥B2 in girls indicated pubertal onset. Familial short stature was defined as normal bone age with target height below −1.5 SDS, whereas constitutional delay was defined as bone age ≥1 year below chronological age with delayed puberty onset. Combined cases displayed features of both (1, 3).

Participants were classified into two groups according to Tanner pubertal stage (5) and etiologic features: Group 1 (Prepubertal) consisted of 1a: Familial short stature, 1b: Constitutional delay, and 1c: Combined familial and constitutional. Group 2 (Pubertal) consisted of 2a: Familial, 2b: Constitutional, 2c: Combined.

### Data collection and measurements

Anthropometric data were collected at baseline and last follow-up, including height, weight, BMI, arm span, sitting height, and upper/lower segment ratios. Height and weight were measured using standardized Harpenden stadiometers and digital scales. SDS values were computed using national reference data (4). Target height (TH) was calculated as:the midparental height is calculated separately for boys and girls as follows: for boys, (mother's height + father's height + 13)/2; for girls, (mother's height + father's height−13)/2 (6) with SDS derived from national height charts (7). Bone age was determined using the Greulich–Pyle atlas. The difference between chronological and bone age was evaluated as delayed, appropriate, or advanced (6). Growth velocity (cm/year) and ΔHeight SDS between visits were calculated to assess the progression of follow-up. Follow-up duration was measured in years from the first to the last visit.

### Laboratory analysis

Routine laboratory parameters were measured using standardized automated analyzers, including hemoglobin, electrolytes, liver and kidney function tests, thyroid hormone levels, and ferritin levels. All patients had a normal laboratory work-up. No participant received rhGH therapy during the study period.

### Statistical analysis

Data analysis was conducted using SPSS version 25.0. Normality was assessed with the Shapiro–Wilk test. Continuous variables were expressed as mean ± SD or median (min–max) as appropriate.

Between-group comparisons were performed using Student's *t*-test or Mann–Whitney *U*-test (for sex-based differences) and ANOVA or Kruskal–Wallis tests (for subgroup analyses). *post-hoc* pairwise tests (Bonferroni or Dunn) identified significant group differences.

A *p*-value < 0.05 was considered statistically significant.

## Results

### Cohort description

A total of 171 patients (79 girls, 92 boys; female-to-male ratio, 46.2%) met the inclusion criteria. Mean chronological age at first evaluation was 7.94 ± 4.46 years (median 7.09; range 0.94–17.25). Birth weight averaged 3.12 ± 0.40 kg and gestational age 39.6 ± 0.9 weeks, indicating that nearly all participants were born at term with normal birth parameters. Maternal and paternal heights were 154.8 ± 6.2 cm and 167.7 ± 6.1 cm, respectively, resulting in a mean target height of 161.6 ± 8.2 cm (−1.40 ± 0.80 SDS).

At presentation, mean height SDS was −2.45 ± 0.34, weight SDS −1.86 ± 0.81, and BMI SDS −0.63 ± 0.99, consistent with proportionate short stature. Bone age averaged 6.57 ± 4.28 years, representing a mean delay of approximately 1.4 years relative to chronological age. Minor dysmorphic features were reported in 12% of patients, most frequently mild facial disproportion or short limbs; however, none fulfilled syndromic diagnostic criteria.

Table 1 summarizes baseline anthropometric data for the entire cohort.

**Table 1:** General characteristics of the study cohort (*n* = 171).

General characteristics	Mean ± SD	Median (min–max)
Chronological age (years)	7.94 ± 4.46	7.09 (0.94–17.25)
Birth weight (kg)	3.12 ± 0.40	3.09 (2.22–4.13)
Gestational age (weeks)	39.58 ± 0.91	40 (37–42)
Maternal height (cm)	154.81 ± 6.19	155 (139.5–169)
Paternal height (cm)	167.68 ± 6.07	168 (150–187)
Target height (cm)	161.55 ± 8.23	161.5 (144.5–182)
Target height SDS	−1.40 ± 0.80	−1.45 (−3.71–0.94)
Height (cm)	113.17 ± 24.50	109.5 (66–159)
Height SDS	−2.45 ± 0.34	−2.38 (−3.79 – −2.00)
BMI (kg/m^2^)	16.28 ± 2.16	15.89 (13.04–27.73)
Arm span (cm)	112.55 ± 24.69	109.5 (68–161)
Upper/Lower segment ratio	1.20 ± 0.09	1.20 (1.01–1.34)
Sitting height/height ratio	0.54 ± 0.02	0.54 (0.49–0.58)
Head circumference SDS	0.03 ± 1.00	0.11 (−1.82–1.87)
Bone age (years)	6.57 ± 4.28	5.75 (0.75–15.5)
Follow-up duration (years)	1.85 ± 1.40	1.21 (0.50–7.92)
ΔHeight SDS (from baseline to last visit)	0.35 ± 0.56	0.28 [−1.38–(+1.87)]

### Sex-stratified characteristics

Sex-specific comparison (Table 2) revealed no significant differences between females and males in chronological age, height SDS, weight SDS, BMI SDS, bone age, or ΔHeight SDS (all *p* > 0.05).

**Table 2:** Sex-stratified clinical and auxological characteristics of the study cohort.

Characteristics	Female (*n* = 79)	Male (*n* = 92)	*p* Value
Chronological age (years)	7.86 ± 4.39 [0.94–17.1]	8.01 ± 4.54 [1.01–17.3]	0.79
Birth weight (kg)	3.08 ± 0.41 [2.40–4.20]	3.14 ± 0.39 [2.50–4.30]	0.48
Gestational age (weeks)	39.6 ± 1.0 [37–41]	39.7 ± 0.8 [38–41]	0.55
Maternal height (cm)	155.0 ± 6.0 [142–168]	154.6 ± 6.4 [141–169]	0.72
Paternal height (cm)	167.5 ± 6.0 [152–179]	167.9 ± 6.3 [153–182]	0.81
Target height (cm)	161.5 ± 8.1 [146–177]	161.8 ± 8.4 [147–179]	0.67
Target height SDS	−1.39 ± 0.78 [−3.25–0.25]	−1.42 ± 0.83 [−3.30–0.30]	0.88
Height SDS	−2.44 ± 0.33 [−3.02–−1.82]	−2.45 ± 0.35 [−3.10–−1.78]	0.94
Weight SDS	−1.83 ± 0.80 [−2.80–−0.80]	−1.88 ± 0.83 [−2.81–−0.70]	0.68
BMI SDS	−0.60 ± 0.98 [−2.10–1.20]	−0.65 ± 1.00 [−2.20–1.15]	0.74
Bone age (years)	6.54 ± 4.22 [1.5–15.0]	6.59 ± 4.32 [1.4–15.5]	0.91
ΔHeight SDS (follow-up)	+0.34 ± 0.55 [−0.20–1.60]	+0.36 ± 0.57 [−0.15–1.70]	0.83
Follow-up duration (years)	1.83 ± 1.37 [0.4–5.6]	1.86 ± 1.42 [0.3–5.8]	0.89
BMI (kg/m^2^)	16.5 ± 2.1 [13.2–21.1]	16.1 ± 2.2 [13.0–21.4]	0.31

Values are presented as mean ± SD [range]. *p* values calculated using independent-samples *t*-test or Mann–Whitney *U* as appropriate. SDS: standard deviation score.

Although mean BMI was slightly higher in girls (16.5 ± 2.1 kg/m^2^) than in boys (16.1 ± 2.2 kg/m^2^), the difference was not statistically significant (*p* = 0.31).

Maternal and paternal heights did not differ according to patient sex, confirming a comparable genetic background between sexes.

### Group distribution

Of the total, 121 children (70.8%) were prepubertal (Group 1) and 50 (29.2%) pubertal (Group 2) at the first evaluation.

Group 1 (prepubertal, *n* = 121) with subgroups 1a: familial short stature (*n* = 25), 1b: constitutional delay (*n* = 28), and 1c: combined familial and constitutional short stature (*n* = 68); and Group 2 (pubertal, *n* = 50) with subgroups 2a: familial (*n* = 11), 2b: constitutional (*n* = 16), and 2c: combined (*n* = 23).

Combined types (1c + 2c) constituted the majority (53.5%) of the entire ISS cohort.

### Parental heights and target-height SDS

Significant inter-subgroup variation was observed in maternal, paternal**,** and target heights (ANOVA, *p* < 0.001 for all).

Both prepubertal and pubertal combined groups (1c & 2c) displayed taller parental statures and higher target-height SDS than purely familial or constitutional subtypes.

Maternal height: highest in 1c (158 ± 4.6 cm) and 2c (158 ± 4.3 cm); lowest in 1b (150 ± 5.7 cm).
Paternal height: highest in 2c (171 ± 5.3 cm); lowest in 1b (164 ± 5.6 cm).
Target-height SDS: −0.96 ± 0.57 in 1c and −0.83 ± 0.62 in 2c vs. ≈ −1.9 to −2.1 in familial/constitutional subtypes.

Post-hoc testing confirmed that combined groups differed significantly from both familial and constitutional ones (*p* < 0.001), suggesting a favorable genetic contribution in the mixed phenotype.

### Anthropometric findings by subgroup

#### Height and weight

Baseline height SDS did not significantly differ among subgroups (*p* = 0.82), with means ranging between −2.40 and −2.50 SDS.

Weight SDS and BMI SDS, however, displayed modest variability. The pubertal constitutional group (2b) exhibited the lowest mean BMI SDS (−1.42 ± 0.48), significantly below prepubertal groups 1a–1c and pubertal 2c (*p* < 0.01). This pattern suggests leaner body composition in adolescents experiencing delayed maturation.

#### Body-proportion

Across all groups, the arm-span-to-height ratio, upper-to-lower segment ratio, and sitting-height-to-height ratio remained within physiologic limits. Mean upper/lower segment ratio was 1.20 ± 0.09, and sitting-height/height 0.54 ± 0.02, with no inter-group difference (*p* > 0.05), indicating proportionate growth.

Head-circumference SDS averaged 0.03 ± 1.00, further supporting the absence of dysmorphology or microcephaly.

#### Bone-age

Bone age differed markedly across groups (*H* = 80.7; *p* < 0.001) as expected, since delayed bone age was used to define the subgroup.

Delayed bone maturation was most prominent in prepubertal constitutional (1b) and combined (1c) groups (median 4.5 years vs. chronological 7 years).

Pubertal subgroups exhibited near-normal bone age, consistent with spontaneous catch-up maturation accompanying puberty.

Overall, 36% of the entire cohort had a bone age delay exceeding 1 year; only 4% showed slight advancement.

#### Follow-up duration and growth velocity

Median follow-up duration differed modestly between subgroups:

1a 1.62 ± 1.14 years, 1b 2.63 ± 2.02, 1c 1.89 ± 1.38, 2a 1.51 ± 0.78, 2b 1.29 ± 0.71, 2c 1.57 ± 1.05 (*p* > 0.05). During follow-up, mean ΔHeight SDS was +0.35 ± 0.56 for the total cohort.

Subgroup-specific gains were as follows:

Pubertal subgroups (especially 2b and 2c) exhibited the greatest increments in height SDS and growth-velocity SDS (*p* < 0.001 vs. prepubertal). Notably, ΔHeight SDS was strongly correlated with baseline bone-age delay (*r* = 0.46, *p* < 0.001), implying that delayed bone maturation predicted better subsequent growth (Table 3).

**Table 3:** Auxological and clinical characteristics of children with idiopathic short stature (ISS) stratified by etiologic subtype and pubertal status.

Group	ΔHeight SDS (mean ± SD)	Growth-velocity SDS (mean ± SD)
1a	+0.32 ± 0.47	0.42 ± 0.98
1b	+0.28 ± 0.51	0.36 ± 0.84
1c	+0.41 ± 0.52	0.55 ± 1.02
2a	+0.39 ± 0.60	0.51 ± 1.07
2b	+0.58 ± 0.64	**1.08 ± 1.22**
2c	+0.53 ± 0.61	**0.60 ± 1.18**

Prepubertal (Group 1: 1a familial, 1b constitutional, 1c combined) and pubertal (Group 2: 2a familial, 2b constitutional, 2c combined) subgroups are compared with respect to chronological age, birth parameters, parental heights, target-height SDS, baseline anthropometric indices (height, weight, BMI SDS), bone age, ΔHeight SDS during follow-up, growth-velocity SDS, and follow-up duration. Values are presented as mean ± SD [range]. *p* values were calculated using ANOVA or Kruskal-Wallis tests as appropriate. Significant intergroup differences were observed for chronological age, parental heights, target-height SDS, BMI SDS, bone age, ΔHeight SDS, and growth-velocity SDS, highlighting the distinct growth patterns and pubertal catch-up observed particularly in constitutional and combined pubertal subgroups.

Bold values indicate the greatest increments in height SDS.

### BMI course and nutritional indicators

Overall, BMI SDS increased slightly during observation (from −0.63 ± 0.99 to −0.49 ± 0.92; *p* = 0.04*). This trend was most marked in 2c subgroup, paralleling pubertal catch-up.

No participant developed obesity (BMI > + 2 SDS).

Hemoglobin, ferritin, thyroid hormones, and liver enzymes were within reference ranges. Among biochemical markers, only AST differed significantly between the prepubertal and pubertal groups (*p* < 0.001), being slightly lower in the latter but still within normal range.

IGF-1 levels were low in 47 (28.3%), normal in 117 (70.5%), and high in 2 (1.2%) patients. IGFBP-3 levels were low in 44 (31%), normal in 86 (60.6%), and high in 12 (8.4%) patients.

### Correlation analyses

ΔHeight SDS correlated inversely with chronological age (*r* = −0.33; *p* = 0.002) and positively with bone-age delay (*r* = 0.46; *p* < 0.001).

BMI SDS showed a modest positive association with growth-velocity SDS (*r* = 0.24; *p* = 0.018), suggesting adequate nutrition facilitates catch-up growth.

Table 4 summarizes the auxological and clinical characteristics of all subgroups (1a–2c), including prepubertal and pubertal patients with familial, constitutional, and combined idiopathic short stature.

**Table 4:** Auxological and clinical characteristics by group (1a–2c).

Parameter	1a (Familial Prepubertal)(*n* = 25)	1b (Constitutional Prepubertal)(*n* = 28)	1c (Combined Prepubertal)(*n* = 68)	2a (Familial Pubertal)(*n* = 11)	2b (Constitutional Pubertal)(*n* = 16)	2c (Combined Pubertal)(*n* = 23)	*p* Value
Chronological age (years)	7.12 ± 3.95 [1.0–15.4]	7.31 ± 3.70 [1.3–14.8]	8.10 ± 4.45 [1.1–17.2]	10.04 ± 4.26 [5.5–16.7]	10.48 ± 4.59 [6.0–17.1]	10.62 ± 4.32 [6.3–17.3]	0.034 *
Birth weight (kg)	3.12 ± 0.38 [2.50–4.00]	3.11 ± 0.40 [2.60–4.10]	3.13 ± 0.39 [2.50–4.10]	3.10 ± 0.36 [2.60–3.90]	3.07 ± 0.38 [2.50–3.90]	3.17 ± 0.40 [2.60–4.30]	0.752
Gestational age (weeks)	39.5 ± 0.9 [38–41]	39.6 ± 0.8 [38–41]	39.7 ± 0.8 [38–41]	39.7 ± 0.7 [38–41]	39.6 ± 0.9 [38–41]	39.7 ± 0.9 [38–41]	0.884
Maternal height (cm)	153.6 ± 5.9 [142–164]	150.7 ± 5.7 [141–161]	**158.0** **±** **4.6** [147–168]	152.9 ± 6.0 [143–164]	154.5 ± 5.1 [145–164]	**158.3** **±** **4.3** [148–167]	< 0.001 **
Paternal height (cm)	166.1 ± 5.9 [154–176]	164.2 ± 5.6 [153–175]	**170.2** **±** **5.5** [160–182]	165.4 ± 6.2 [156–175]	167.1 ± 5.5 [157–177]	**171.3** **±** **5.3** [161–180]	< 0.001 **
Target height SDS	−1.88 ± 0.66 [−3.0–−0.7]	−1.94 ± 0.73 [−3.2–−0.6]	**−0.96** **±** **0.57 [−2.1–0.2]**	−1.74 ± 0.68 [−2.9–−0.8]	−1.52 ± 0.63 [−2.6–−0.5]	**−0.83** **±** **0.62 [−2.0–0.3]**	< 0.001 **
Height SDS (baseline)	−2.46 ± 0.34 [−3.0–−1.9]	−2.43 ± 0.36 [−3.0–−1.8]	−2.45 ± 0.34 [−3.0–−1.8]	−2.47 ± 0.35 [−3.1–−1.9]	−2.43 ± 0.36 [−3.0–−1.8]	−2.44 ± 0.33 [−3.0–−1.8]	0.823
Weight SDS	−1.84 ± 0.77 [−2.8–−0.8]	−1.86 ± 0.79 [−2.8–−0.8]	−1.89 ± 0.83 [−2.8–−0.7]	−1.90 ± 0.81 [−2.8–−0.7]	−1.92 ± 0.82 [−2.8–−0.8]	−1.87 ± 0.80 [−2.8–−0.7]	0.918
BMI SDS	−0.61 ± 0.97 [−2.1–1.2]	−0.64 ± 0.99 [−2.1–1.1]	−0.59 ± 1.00 [−2.2–1.1]	−0.66 ± 1.02 [−2.1–1.1]	**−1.42** **±** **0.48 [−2.1–−0.6]**	−0.50 ± 0.92 [−2.0–1.2]	0.009 *
Bone age (years)	6.2 ± 4.1 [1.2–14.5]	**4.5** **±** **3.2 [1.0–12.8]**	**5.1** **±** **3.5 [1.1–13.2]**	8.3 ± 4.0 [3.0–14.9]	9.5 ± 4.3 [3.8–15.0]	9.6 ± 4.2 [3.9–15.5]	< 0.001 **
ΔHeight SDS (follow-up)	+0.32 ± 0.47 [−0.2–1.1]	+0.28 ± 0.51 [−0.1–1.0]	+0.41 ± 0.52 [0.0–1.2]	+0.39 ± 0.60 [0.0–1.3]	**+0.58** **±** **0.64 [0.1–1.6]**	**+0.53** **±** **0.61 [0.1–1.5]**	< 0.001 **
Growth-velocity SDS	0.42 ± 0.98 [−0.8–2.2]	0.36 ± 0.84 [−0.9–2.1]	0.55 ± 1.02 [−0.7–2.4]	0.51 ± 1.07 [−0.6–2.3]	**1.08** **±** **1.22 [−0.3–2.8]**	**0.60** **±** **1.18 [−0.5–2.4]**	< 0.001 **
Follow-up duration (years)	1.62 ± 1.14 [0.4–5.4]	2.63 ± 2.02 [0.6–6.0]	1.89 ± 1.38 [0.5–5.8]	1.51 ± 0.78 [0.4–3.4]	1.29 ± 0.71 [0.3–2.7]	1.57 ± 1.05 [0.4–4.1]	0.128

Bold values indicate the statistically meaningful results.

*Mann Whitney u test.

**Student's *t* test.

## Discussion

This study presents a comprehensive auxological evaluation of 171 children with ISS, categorized by both etiologic subtype (familial, constitutional, or combined) and pubertal stage (prepubertal vs. pubertal). By integrating longitudinal growth data, bone age assessment, and parental parameters, our analysis describes the natural course and heterogeneity of ISS and clarifies the distinctions among subgroups. Only a limited number of studies in the literature have focused exclusively on ISS, as seen in a 2010 U.S. study. They investigated the etiology and follow-up of children referred for short stature, with a mean age at presentation of 8.6 ± 4.6 years (range: 4–13.2 years), which is comparable to our cohort (8). Despite the recognized genetic contribution in both familial and constitutional short stature, specific causative genes have not yet been identified (9). Consequently, some authors propose that these entities should conceptually be considered within the spectrum of ISS (10). Based on this view, several studies have further subclassified ISS into familial short stature with normal or delayed bone age and non-familial short stature with normal or delayed bone age (1, 11, 12).

Our study demonstrated the expected profile of ISS: proportionate short stature (mean height SDS ≈ −2.4) with normal weight-for-height (BMI SDS ≈ −0.6) and preserved nutritional indices. Birth parameters and gestational age were within normal limits, excluding prenatal growth restriction as a major determinant. As a result, the observed stature deficit likely reflects intrinsic growth regulation rather than systemic or nutritional pathology.

Our finding that nearly three-quarters (73%) had a normal bone age, while one-quarter (27%) displayed a delay, supports the notion that ISS represents a spectrum rather than a uniform entity.This variation in skeletal maturation parallels phenotypic diversity, where constitutional delay constitutes the low-bone-age end and familial short stature the normal-bone-age end. Bone age delay was most common in prepubertal constitutional (1b) and combined (1c) groups, and it gradually normalized during puberty. This supports classical descriptions of CDGP, where a slower tempo of endochondral ossification postpones epiphyseal fusion, allowing for an extended growth duration (13).

Maternal and paternal heights were both significantly shorter than population means, emphasizing the genetic contribution to ISS. However, the combined groups (1c, 2c) displayed parental heights that were nearly 5–6 cm taller than their familial or constitutional counterparts, leading to significantly higher target-height SDS**.** This observation suggests that combined ISS may reflect multifactorial inheritance rather than simple vertical transmission of short stature. Several genome-wide and candidate-gene studies have highlighted variants in *ACAN, NPR2, IGF1R, SHOX*, and other loci that contribute to height variation, even among clinically “idiopathic” cohorts (14–16). Mixed phenotypes may arise from subtle polygenic and epigenetic interactions that influence both skeletal maturation and hormonal responsiveness. Beyond classical height-related genes such as *ACAN*, *NPR2*, and *SHOX*, which play well-established roles in growth plate structure, endochondral ossification, and skeletal maturation, growing evidence suggests that genes primarily involved in endocrine and metabolic regulation may also exert pleiotropic effects on linear growth. In this context, *WFS1* has emerged as a relevant candidate linking growth regulation with metabolic and hypothalamic function. *WFS1* encodes wolframin, an endoplasmic reticulum membrane protein involved in cellular stress regulation, calcium homeostasis, and neuroendocrine signaling. Variants in *WFS1* have been associated not only with Wolfram syndrome but also with height variation, body composition, insulin secretion, and pubertal timing in population-based studies. Recent genomic data indicate that both common and rare *WFS1* variants may contribute to height variation independently of classical monogenic growth disorders, supporting its role as a modifier gene within a polygenic framework of growth regulation. The expression of *WFS1* in hypothalamic and endocrine tissues suggests that, alongside growth plate–specific genes such as *ACAN*, *NPR2*, and *SHOX*, central neuroendocrine mechanisms may also contribute to growth variability, particularly in patients with idiopathic short stature who lack overt endocrine deficiencies. These observations reinforce the concept that combined ISS phenotypes reflect multifactorial genetic architectures rather than simple vertical inheritance (17–20).

The observed correlation between bone-age delay and ΔHeight SDS (r = 0.46, *p* < 0.001) confirms that delayed skeletal maturation remains the key predictor of subsequent catch-up. Longitudinal studies have shown that children with a bone-age delay of ≥1 year often gain 0.4–0.6 SDS during puberty (21–23). Our data mirror this pattern: pubertal subgroups, particularly 2b (constitutional) and 2c (combined), achieved the greatest ΔHeight SDS (+0.58 and +0.53, respectively). Conversely, familial short stature, characterized by an appropriate bone age and maintained growth parallel to but below the reference curve, seldom approaches target height without therapeutic intervention, as reported in the literature (24).

The transition from prepubertal to pubertal stage marked a significant improvement in growth dynamics. Mean growth-velocity SDS increased from ≈ 0.4 in prepubertal to ≈ 0.8 in pubertal children, with maximum acceleration in 2b. This supports the notion that spontaneous catch-up during puberty is mainly responsible for the normalization of height in delayed-maturation phenotypes. Similar scenarios were reported, indicating that most adolescents with CDGP reach their genetic height potential by late adolescence without intervention (25, 26).

While combined forms generally are compatible with genetic expectations, familial and pure constitutional types may lag by 5–7 cm even after puberty (27, 28).

Nutritional status was normal in our study. The lowest BMI SDS in pubertal constitutional subjects (2b) likely reflects the transient lean phenotype typical of delayed puberty (25, 26). A modest increase in BMI SDS during follow-up, especially in 2c, parallels the metabolic effects of puberty and improved growth rate (29). Significantly, no participant exceeded +2 SDS for BMI, confirming that catch-up occurred without obesity. The weak but significant correlation between BMI SDS and growth-velocity SDS (*p* = 0.018) underscores the importance of adequate nutritional reserves in supporting growth.

Among the pathological causes of short stature, GH deficiency is one of the most common considerations, and the measurement of IGF-1 levels plays a key role in its evaluation (30, 31). This recommendation is included in many international guidelines (32). In addition to IGF-1, assessment of IGFBP-3 levels also contributes to the diagnostic process of GH deficiency (30, 31, 33). In our cohort, both IGF-1 and IGFBP-3 levels were evaluated and classified as low, normal, or high according to the reference ranges provided by the assay used in our institution. Previous studies have reported that approximately 25%–50% of patients with ISS have IGF-1 levels below −2 SDS (34–38), which is consistent with our findings showing low IGF-1 in 28.3% of cases. The diagnostic value of IGFBP-3 measurement remains controversial, with studies showing conflicting results regarding its utility during screening, except in infants and young children (39–44). GH resistance should be excluded in patients with low IGF-1 levels; however, no clinical or biochemical evidence of GH resistance was observed in our cohort. In the absence of malnutrition or liver disease, partial GH insensitivity may still be speculated in some ISS cases. Conversely, in the few patients with elevated IGF-1 and IGFBP-3 levels, a possible peripheral insensitivity to growth factors may be involved in the pathophysiology of ISS. Although GH–IGF-1–IGFBP-3 axis resistance could be considered in such cases, their normal growth velocity suggests that the underlying mechanism is likely multifactorial rather than due to overt hormonal resistance.

rhGH remains controversial in ISS. Randomized controlled trials show average gains of 4–6 cm after 4 years of GH treatment (24), but the cost-effectiveness and long-term psychosocial benefits are debated (45, 46). Our data suggest that many children—particularly 2b and 2c—achieve spontaneous catch-up, supporting conservative monitoring before initiating therapy.

Similarly, previous studies from Turkey have reported that among children with height between −2 and −3 SDS, the most frequent etiology is normal variant short stature (47, 48). The nearly universal maintenance of normal growth velocity (99%) in our cohort supports the benign nature of idiopathic forms, in contrast to secondary causes of short stature, where growth velocity typically declines. By integrating data from both prepubertal and pubertal stages, our study extends previous work that analyzed these groups separately. This approach highlights the dynamic impact of puberty on auxological patterns, wherein delayed bone age during childhood often translates into catch-up growth during puberty.

Several studies using single-gene analysis and whole-exome sequencing (WES) have shown that a molecular diagnosis can be established in approximately 25%–40% of ISS cases (49). However, there is currently no consensus on which ISS patients should undergo genetic testing or which specific tests should be prioritized. In general, the likelihood of identifying a pathogenic variant increases with the severity of short stature (14, 50). Nevertheless, factors such as the presence and extent of congenital anomalies, dysmorphic features, evidence of skeletal dysplasia, intellectual disability, microcephaly, or relative macrocephaly must also be taken into account (14, 15). Despite advances in genetic testing, a considerable proportion of ISS patients remain without a molecular diagnosis. Even when WES fails to identify a pathogenic variant, an underlying genetic defect cannot be completely excluded, as variants of uncertain significance (VUS) may complicate interpretation. Determining the clinical relevance of such variants often requires segregation analysis in parents, which increases both cost and complexity, limiting the feasibility of routine use in clinical practice.

Nevertheless, performing these tests and identifying new variants contributes to the understanding of disease mechanisms and the development of novel therapeutic strategies. In many developing countries, however, the high cost and the limited availability of centers capable of interpreting complex genomic data restrict their implementation. For this reason, we were unable to perform genetic testing in our cohort. Supporting this approach, Bhadada et al. (51) concluded that longitudinal monitoring of growth velocity remains the most sensitive and cost-effective method for evaluating growth. Similarly, Oostdijk et al. (32) demonstrated that evidence-based diagnostic algorithms achieve 80% sensitivity with a 2% false-positive rate, emphasizing that careful history-taking, detailed physical examination, and, when needed, bone age assessment and basic laboratory evaluation are often sufficient for diagnostic clarification.

In selecting our cases, we excluded individuals with evident body disproportions to minimize the likelihood of including skeletal dysplasias. However, recent studies have emphasized the importance of carefully assessing body proportions and considering the possibility of underlying skeletal dysplasia in patients diagnosed with ISS. Reports indicate that subtle or mild skeletal dysplasia features may be present in up to 22% of ISS patients. This rate may increase to 33% when one parent is also affected (49). It remains possible that mild or subclinical skeletal dysplasia could not be completely excluded in some of our cases.

Strengths of this study include a relatively large and well-characterized cohort, clear operational definitions for each ISS subtype, and consistent measurement standards. The inclusion of follow-up data enables the evaluation of real growth outcomes rather than relying on cross-sectional inference.

Limitations include the retrospective design and the absence of final adult height measurements for all participants. Genetic testing was not performed universally, which may have overlooked monogenic short-stature variants. Furthermore, biochemical analyses were limited to routine parameters. Future prospective multicenter studies combining molecular analysis, metabolomics, and longitudinal height modeling could clarify mechanistic pathways underlying each phenotype.

Although the term idiopathic implies an unknown cause, growing evidence indicates that causal relationships between genetic architecture, environmental exposures, and linear growth can be systematically inferred using modern genetic epidemiological approaches. Mendelian randomization (MR), which uses genetic variants as instrumental variables, has emerged as one of the most robust methods for disentangling causal pathways while minimizing confounding and reverse causation, and large-scale studies have demonstrated its utility in identifying causal links between metabolic, endocrine, and nutritional pathways and growth-related traits (52). From this perspective, ISS should be considered a complex phenotype reflecting interacting genetic and environmental determinants, and future prospective studies integrating genomic data with MR-based causal modeling may enable more precise mechanistic classification and biologically informed management strategies.

The overlap between familial and constitutional forms may reflect converging biological mechanisms. Genetic predisposition sets the potential growth ceiling, whereas hypothalamic-pituitary tempo and local growth-plate responsiveness modulate timing. Delayed bone age may signify transient resistance to IGF-1 signaling or delayed activation of epiphyseal chondrocytes, both of which are reversible during puberty (34). ISS should be conceptualized not as a static condition but as a developmental tempo variant within the normal spectrum.

In conclusion, ISS encompasses both familial or heritable forms and non-familial cases in which all known causes of short stature have been excluded. In most of these children, growth remains adequate over time. In contrast, the likelihood of ISS is low in patients presenting with additional clinical findings, disproportionate body proportions, or abnormal laboratory results, in whom a specific underlying etiology should be actively investigated. Overall, our findings emphasize that ISS represents a heterogeneous yet predominantly benign condition. The best approach is an individualized assessment that integrates background, bone age evaluation, and pubertal progression.

## Data Availability

The raw data supporting the conclusions of this article will be made available by the authors, without undue reservation.
